# Supplementary material for: Comparative Analysis of mRNA and miRNA Expression between Dermal Papilla Cells and Hair Matrix Cells of Hair Follicles in Yak
Source: Cells. 2022 Dec 9;11(24):3985. doi: 10.3390/cells11243985 (PMC9776824; doi:10.3390/cells11243985)
Supplement: Supplementary file 1 [file cells-11-03985-s001.zip › Table S1 Primers used in qPCR.pdf]

Table S1 Primers used in qPCR

Name	Accession number	Forward primer	Reverse primer	Product size
SFRP1	ENSBGRG00000011484	GAGATGCTCAAGTGCGACAAG	TTTCATCCTCAGGGCAAACTC	180
BCL2	ENSBGRG00000019339	GGGGTCATGTGTGTGGAGAG	TCCACAAAGGCGTCCCAG	137
WNT5A	ENSBGRG00000021892	ATTCTTGGTGGTCGCTAGGT	ACTCCTTGATGCCCGTCTTG	180
PCOLCE	ENSBGRG00000020493	AGACCCCCAACTACACCAGA	TCTGGCTCTTAGGGACCGTT	144
TGFB2	ENSBGRG00000005633	TCTCCTGCTAATGTTGTTGCC	AATGTAAAGTGGGCGTAGGC	133
ITGA6	ENSBGRG00000010970	CAACAAAGACGGGTGGCAAG	AGACGAATCGGCTTGACGTT	132
LEF1	ENSBGRG00000006944	TGTCAACTCCAAACAAGGCAT	ATACACAGGCTGACCTTGCC	127
SOX9	ENSBGRG00000026479	CTCTGGAGACTGCTGAACGA	GCGGCTGGTACTTGTAGTCC	106
bta-miR-143	MI0009743	TGAGATGAAGCACTGTAGCTCG		
bta-miR-199a-3p	MI0004758	ACAGTAGTCTGCACATTGGTTA		
bta-miR-214	MI0005040	ACAGCAGGCACAGACAGGCAGT		
bta-miR-218	MI0009780	TTGTGCTTGATCTAACCATGTG		
bta-miR-200a	MI0005037	TAACACTGTCTGGTAACGATGTT		
bta-miR-205	MI0004759	TCCTTCATTCCACCGGAGTCTG		
bta-miR-31	MI0004762	AGGCAAGATGCTGGCATAGCT		
pri-miR-143	ENSBTAT00000042493.3	CGGGAAGGGAGAAGTTGTT	TTCCTCAGCGGACATTCTG	120
pri-miR-199a	ENSBTAT00000042310.2	GCCCCAGTGTTCAGACTAC	ACAAATGTCTTCCTCCTTGG	137
pri-miR-200a	ENSBTAT00000042377.2	GGACAGTGCTGGATTTCTCG	CTGTTTGCCTGCCCCTAGT	159
pri-miR-205	ENSBTAT00000042233.2	GCTTCTCTTGTCCTTCATTCC	CACTGAGCCTTTCCTTTTCC	138
pri-miR-31	ENSBTAT00000063739.2	CTCAGGTGGAAGGGAAGATT	CATAGCAGGTTCGCAGTTC	123
