# Supplementary material for: Comparative Analysis of mRNA and miRNA Expression between Dermal Papilla Cells and Hair Matrix Cells of Hair Follicles in Yak
Source: Cells. 2022 Dec 9;11(24):3985. doi: 10.3390/cells11243985 (PMC9776824; doi:10.3390/cells11243985)
Supplement: Supplementary file 1 [file cells-11-03985-s001.zip › Table S2 Information of reads mapping to the reference genome.pdf]

Table S2. Information of reads mapping to the reference genome

Sample	Clean bases	Raw reads	Clean reads	Total mapped reads
DPCs1	7.08G	49.06M	48083610	44716378(93.00%)
DPCs2	7.00G	48.80M	47867468	44473541(92.91%)
DPCs3	7.00G	48.55M	47591316	44203481(92.88%)
HMCs1	7.24G	50.05M	49097296	45272937(92.21%)
HMCs2	7.37G	51.03M	50057282	46112250(92.12%)
HMCs3	7.32G	50.67M	49761244	45798561(92.04%)
