# Supplementary material for: Comparative Analysis of mRNA and miRNA Expression between Dermal Papilla Cells and Hair Matrix Cells of Hair Follicles in Yak
Source: Cells. 2022 Dec 9;11(24):3985. doi: 10.3390/cells11243985 (PMC9776824; doi:10.3390/cells11243985)
Supplement: Supplementary file 1 [file cells-11-03985-s001.zip › Table S3 Information of sRNA after quality filtering and mapped to yak genome and miRbase.pdf]

Table S3 Information of sRNA after quality filtering and mapped to yak genome and miRbase

Samples	RawReads	CleanReads	Aligned_reads in reference genome	Aligned_reads in miRbase
DPCs_1	27.29M	23904796	22977654(96.12%)	17419876(72.87%)
DPCs_2	26.92M	23937085	23053738(96.31%)	17544649(73.29%)
DPCs_3	27.55M	24246764	23354073(96.32%)	17367241(71.63%)
HMCs_1	27.52M	24693655	23672179(95.86%)	18138656(73.45%)
HMCs_2	27.51M	24484528	23422697(95.66%)	17689056(72.25%)
HMCs_3	26.69M	24006933	22984889(95.74%)	17251524(71.86%)
