# Supplementary material for: Comparative Analysis of mRNA and miRNA Expression between Dermal Papilla Cells and Hair Matrix Cells of Hair Follicles in Yak
Source: Cells. 2022 Dec 9;11(24):3985. doi: 10.3390/cells11243985 (PMC9776824; doi:10.3390/cells11243985)
Supplement: Supplementary file 1 [file cells-11-03985-s001.zip › Table S4 Statistics of the counts of known miRNAs and novel miRNAs.pdf]

Table S4. Statistics of the counts of known miRNAs and novel miRNAs

Types	Total	DPCs_1	DPCs_2	DPCs_3	HMCs_1	HMCs_2	HMCs_3
Known miRNAs	757	622	619	618	608	609	623
Novel miRNAs	2096	1069	1099	1106	1142	1166	1196
